# Supplementary material for: Application of Machine Learning to Metabolomic Profile Characterization in Glioblastoma Patients Undergoing Concurrent Chemoradiation
Source: Metabolites. 2023 Feb 17;13(2):299. doi: 10.3390/metabo13020299 (PMC9961856; doi:10.3390/metabo13020299)
Supplement: Supplementary file 1 [file metabolites-13-00299-s001.zip › metabolites-2156067-supplementary.pdf]

Supplementary Table S1: Table details metabolites with significant changes. Req rounded to the second decimal place. Sub class information obtained using Metabolomics Workbench (https://www.metabolomicsworkbench.org/databases/refmet/index.php). "NA" lower match score or library match.

BinBase name	PubChem	KEGG	Superclass	rq_new_est	P Value
sorbitol	5780	C00794	Organic oxygen compounds	-1.744103192	3.03E-18
glucuronic acid	94715	C00191	Organic oxygen compounds	-1.661830824	4.37E-20
mannose-6-phosphate	65127	C00275	Organic oxygen compounds	-1.659637299	9.02E-13
mannitol	6251	C00392	Organic oxygen compounds	-1.517557588	5.54E-19
linoleic acid	5280450	C01595	Lipids and lipid-like molecules	-1.37343669	1.64E-08
linolenic acid	5280934	C06427	Lipids and lipid-like molecules	-1.331943426	7.14E-09
6-deoxyglucitol	151266		Organic oxygen compounds	-1.206081692	1.32E-08
3-hydroxybutyric acid	92135	C01089	Organic acids and derivatives	-0.924644368	0.000100824
gluconic acid	6857417	C00800	Organic acids and derivatives	-0.704779634	0.016795591
tocopherol gamma-	92729	C02483	Lipids and lipid-like molecules	-0.70258476	0.000120132
oleic acid	445639	C00712	Lipids and lipid-like molecules	-0.603480924	0.00038113
glycerol	753	C00116	Organic oxygen compounds	-0.533083884	0.015413684
2-hydroxybutanoic acid	440864	C05984	Organic acids and derivatives	0.270067871	0.013981071
N-acetylglycine	10972	C02055	Organic acids and derivatives	0.29920216	0.040657137
hydroxyproline dipeptide	61159526		Organic acids and derivatives	0.382516798	0.041324664
oleamide	5283387	C19670	Lipids and lipid-like molecules	0.40541256	1.53E-06
tocopherol alpha-	14985	C02477	Lipids and lipid-like molecules	0.409796305	0.01515781
uric acid	1175	C00366	Organoheterocyclic compounds	0.410680566	0.001413049
arachidic acid	10467	C06425	Lipids and lipid-like molecules	0.474019778	0.004042598
threonic acid	5460407	C01620	Organic oxygen compounds	0.492712598	4.33E-05
3-aminoisobutyric acid	64956	C05145	Organic acids and derivatives	0.525882779	0.014904055
indole-3-propionic acid	3744		Organoheterocyclic compounds	0.549573137	0.000163273
squalene	638072	C00751	Lipids and lipid-like molecules	0.565632998	2.41E-06
2-deoxytetronic acid	150929		Organic acids and derivatives	0.56792359	1.25E-05
inosine	6021	C00294	Nucleosides, nucleotides, and analogues	0.572993734	0.016102833
aconitic acid	643757	C00417	Organic acids and derivatives	0.58031309	0.001087993
5-methoxytryptamine	1833	C05659	Organoheterocyclic compounds	0.588423889	0.000315391
pseudo uridine	15047	C02067	Nucleosides, nucleotides, and analogues	0.592419727	0.000116934
glutamic acid	33032	C00025	Organic acids and derivatives	0.607066458	0.000421518
palmitic acid	985	C00249	Lipids and lipid-like molecules	0.610786018	0.000361021
pipecolinic acid	6931662	C00408	Organic acids and derivatives	0.616464079	6.43E-05

phosphoethanolamine	1015	C00346	Organic acids and derivatives	0.64565875	0.000195703
erythritol	222285	C00503	Organic oxygen compounds	0.6510168	9.32E-05
xylitol	6912	C00379	Organic oxygen compounds	0.655524549	0.000464126
glycerol-alpha-phosphate	754	C03189	Lipids and lipid-like molecules	0.657325322	1.02E-05
adenosine	60961	C00212	Nucleosides, nucleotides, and analogues	0.66071442	0.000271334
2,3-dihydroxybutanoic acid	250402		Organic oxygen compounds	0.673687123	5.81E-05
arachidonic acid	444899	C00219	Lipids and lipid-like molecules	0.681398423	0.000190558
inositol-4-monophosphate	440043	C03546	Organic oxygen compounds	0.68448704	0.003944813
3-Aminopiperidine-2,6-dione	134508		Organic acids and derivatives	0.696733034	0.00128681
xanthine	1188	C00385	Organoheterocyclic compounds	0.700183044	1.89E-06
levoglucosan	2724705	C22350	Organoheterocyclic compounds	0.702005579	6.80E-07
myristic acid	11005	C06424	Lipids and lipid-like molecules	0.710294654	2.67E-05
glyceric acid	752	C00258	Organic oxygen compounds	0.712553047	0.000362198
2-ethylcaproic acid	8697		Lipids and lipid-like molecules	0.72635205	9.19E-05
isocitric acid	5318532	C00451	Organic acids and derivatives	0.726616127	2.20E-06
citric acid	311	C00158	Organic acids and derivatives	0.748033739	0.000392481
aspartic acid	5960	C00049	Organic acids and derivatives	0.755238702	2.08E-06
myo-inositol	892	C00137	Organic oxygen compounds	0.769032442	0.000371229
6-deoxyglucose	441480	C08352	Organic oxygen compounds	0.782550078	1.51E-07
guanosine	6802	C00387	Nucleosides, nucleotides, and analogues	0.787471242	0.00063282
triethanolamine	7618	C06771	Organic nitrogen compounds	0.787834845	0.000381666
heptanoic acid	8094	C17714	Lipids and lipid-like molecules	0.798841483	0.00022587
2-hydroxyglutaric acid	43	C02630	Organic acids and derivatives	0.801325561	3.54E-05
proline	145742	C00148	Organic acids and derivatives	0.802153852	0.000504163
deoxypentitol	270738		Organic oxygen compounds	0.814554933	3.45E-08
9-myristoleate	5461014		Lipids and lipid-like molecules	0.815553083	0.000335399
indoxyl sulfate	10258		Organic acids and derivatives	0.833307239	7.15E-08
capric acid	2969	C01571	Lipids and lipid-like molecules	0.833860879	1.58E-06
caproic acid	8892	C01585	Lipids and lipid-like molecules	0.834181737	0.000226587
ribitol	827	C00474	Organic oxygen compounds	0.83902516	1.53E-05
ribose	5779	C00121	Organic oxygen compounds	0.855628861	4.23E-05
methanolphosphate	13130		Organic acids and derivatives	0.874438614	4.00E-07
propyleneglycol	259994	C02912	Organic oxygen compounds	0.893100673	3.01E-05
mandelic acid	1292		Benzenoids	0.89458187	8.62E-05
fumaric acid	444972	C00122	Organic acids and derivatives	0.899176407	7.42E-08
hypoxanthine	790	C00262	Organoheterocyclic compounds	0.90364736	9.29E-07

phenylethylamine	1001	C05332	Benzenoids	0.904517777	4.38E-05
kynurenine	25245862	C01718	Organic oxygen compounds	0.907527954	2.88E-07
ketoisoleucine	47	C03465	Organic acids and derivatives	0.934114142	8.62E-07
adipic acid	196	C06104	Lipids and lipid-like molecules	0.939214724	9.88E-05
trans-4-hydroxyproline	5810	C01157	Organic acids and derivatives	0.955823562	7.78E-08
glycerol-3-galactoside	16048618	C05401	Lipids and lipid-like molecules	0.967414672	2.99E-08
fucose	439650	C02095	Organic oxygen compounds	0.970338203	1.20E-09
indole-3-acetate	802	C00954	Organoheterocyclic compounds	0.972320483	2.99E-08
tartaric acid	444305	C00898	Organic oxygen compounds	0.974513969	8.02E-06
glutamine	5961	C00064	Organic acids and derivatives	0.975585203	3.48E-05
hypotaurine	107812	C00519	Organic acids and derivatives	0.983711393	5.38E-08
threitol	169019	C16884	Organic oxygen compounds	0.992428344	9.34E-09
lignoceric acid	11197	C08320	Lipids and lipid-like molecules	0.995733064	5.43E-06
lactulose	11333	C07064	Organic oxygen compounds	1.023933365	6.15E-08
lactic acid	612	C01432	Organic acids and derivatives	1.024777763	2.80E-06
maltose	439186	C00208	Organic oxygen compounds	1.033187368	3.70E-06
tryptamine	1150	C00398	Organoheterocyclic compounds	1.037870216	5.82E-05
urea	1176	C00086	Organic acids and derivatives	1.046969757	1.70E-06
benzoic acid	243	C00180	Benzenoids	1.051970849	4.87E-08
malic acid	525	C00711	Organic acids and derivatives	1.06377356	7.78E-11
histidine	6274	C00135	Organic acids and derivatives	1.067398638	4.08E-05
succinate semialdehyde	9543238		Lipids and lipid-like molecules	1.073998938	3.37E-06
acetoacetate	6971017	C00164	Organic acids and derivatives	1.080215576	8.54E-07
ethanolamine	700	C00189	Organic nitrogen compounds	1.081110309	2.33E-08
citrulline	9750	C00327	Organic acids and derivatives	1.084149361	1.56E-09
2-hydroxyvaleric acid	98009		Lipids and lipid-like molecules	1.084471601	9.29E-09
2-aminobutyric acid	6657	C02721	Organic acids and derivatives	1.085690211	4.07E-06
beta-alanine	239	C00099	Organic acids and derivatives	1.091216389	2.84E-10
1-monopalmitin	14900		Lipids and lipid-like molecules	1.099380772	4.63E-07
salicylic acid	338	C00805	Benzenoids	1.102073456	2.76E-06
acetaminophen	1983	C06804	Benzenoids	1.107858101	4.57E-06
acetaminophen glucuronide	83944		Organic oxygen compounds	1.117050477	1.22E-06
phenol	996	C00146	Benzenoids	1.118833689	2.47E-07
lauric acid	3893	C02679	Lipids and lipid-like molecules	1.123835216	4.08E-07
isothreonic acid	151152	C00639	Organic oxygen compounds	1.146566802	2.82E-10
azelaic acid	19347555	C08261	Lipids and lipid-like molecules	1.147879337	3.00E-07
cystine	595	C01420	Organic acids and derivatives	1.156164328	7.99E-08
sucrose	5988	C00089	Organic oxygen compounds	1.159878886	8.65E-08
creatinine	588	C00791	Organic acids and derivatives	1.162729574	3.61E-07
serine	5951	C00065	Organic acids and derivatives	1.165318348	5.71E-08

isoleucine	6306	C00407	Organic acids and derivatives	1.18415286	2.40E-08
2-ketobutyric acid	58	C00109	Organic acids and derivatives	1.187938655	6.68E-08
glutaric acid	743	C00489	Organic acids and derivatives	1.189177627	2.92E-07
2-hydroxyisovaleric acid	99823		Lipids and lipid-like molecules	1.197934136	4.12E-10
alpha-ketoglutarate	51	C00026	Organic acids and derivatives	1.200339742	1.14E-08
hippuric acid	464	C01586	Benzenoids	1.204498885	4.80E-08
lyxose	439240	C00476	Organic oxygen compounds	1.217059858	4.57E-08
glycine	750	C00037	Organic acids and derivatives	1.218257591	7.05E-11
conduritol-beta-epoxide	9989541		Organoheterocyclic compounds	1.219341955	3.10E-09
2-ketoisocaproic acid	70	C00233	Organic acids and derivatives	1.221724534	5.70E-10
oxoproline	7405	C01879	Organic acids and derivatives	1.23083697	2.02E-13
lactose	6134	C01970	Organic oxygen compounds	1.251549431	5.30E-12
succinic acid	1110	C00042	Organic acids and derivatives	1.255304045	1.13E-09
3-hydroxypropionic acid	68152	C01013	Organic acids and derivatives	1.271232761	7.73E-12
ribonic acid	5460677	C01685	Organic oxygen compounds	1.288524026	4.92E-10
fructose	439709	C02336	Organic oxygen compounds	1.294867984	9.46E-14
phosphate	1004	C00009	Homogeneous non-metal compounds	1.304785806	7.27E-10
cholesterol	5997	C00187	Lipids and lipid-like molecules	1.314927656	1.18E-11
putrescine	1045	C00138	Organic nitrogen compounds	1.321182842	5.30E-10
leucine	6106	C00123	Organic acids and derivatives	1.326070926	8.32E-11
lactamide	94220		Organic oxygen compounds	1.334167768	8.89E-11
phthalic acid	1017	C01606	Benzenoids	1.361315979	1.38E-08
terephthalic acid	22416364	C06337	Benzenoids	1.390455495	2.07E-10
glucose	64689	C00221	Organic oxygen compounds	1.390578057	3.08E-14
phenylacetic acid	999	C07086	Benzenoids	1.421159264	2.49E-11
xylose	135191	C00181	Organic oxygen compounds	1.428130139	7.58E-14
indole-3-lactate	92904	C02043	Organoheterocyclic compounds	1.433659257	6.61E-12
glycolic acid	757	C00160	Organic acids and derivatives	1.477396417	3.77E-12
alanine	5950	C00041	Organic acids and derivatives	1.489719822	2.86E-10
mannose	18950	C00159	Organic oxygen compounds	1.493799144	1.14E-11
valine	6287	C00183	Organic acids and derivatives	1.497286265	3.55E-12
ornithine	6262	C00077	Organic acids and derivatives	1.499587807	1.75E-13
methionine	6137	C00073	Organic acids and derivatives	1.507823367	2.29E-11
asparagine	6267	C00152	Organic acids and derivatives	1.542920719	4.48E-10
threonine	6288	C00188	Organic acids and derivatives	1.611893927	2.79E-12
lysine	5962	C00047	Organic acids and derivatives	1.631233023	1.51E-13
tyrosine	6057	C00082	Organic acids and derivatives	1.633429276	9.11E-17
phenylalanine	6140	C00079	Organic acids and derivatives	1.786072299	2.74E-17
tryptophan	6305	C00078	Organoheterocyclic compounds	1.830114175	2.78E-18

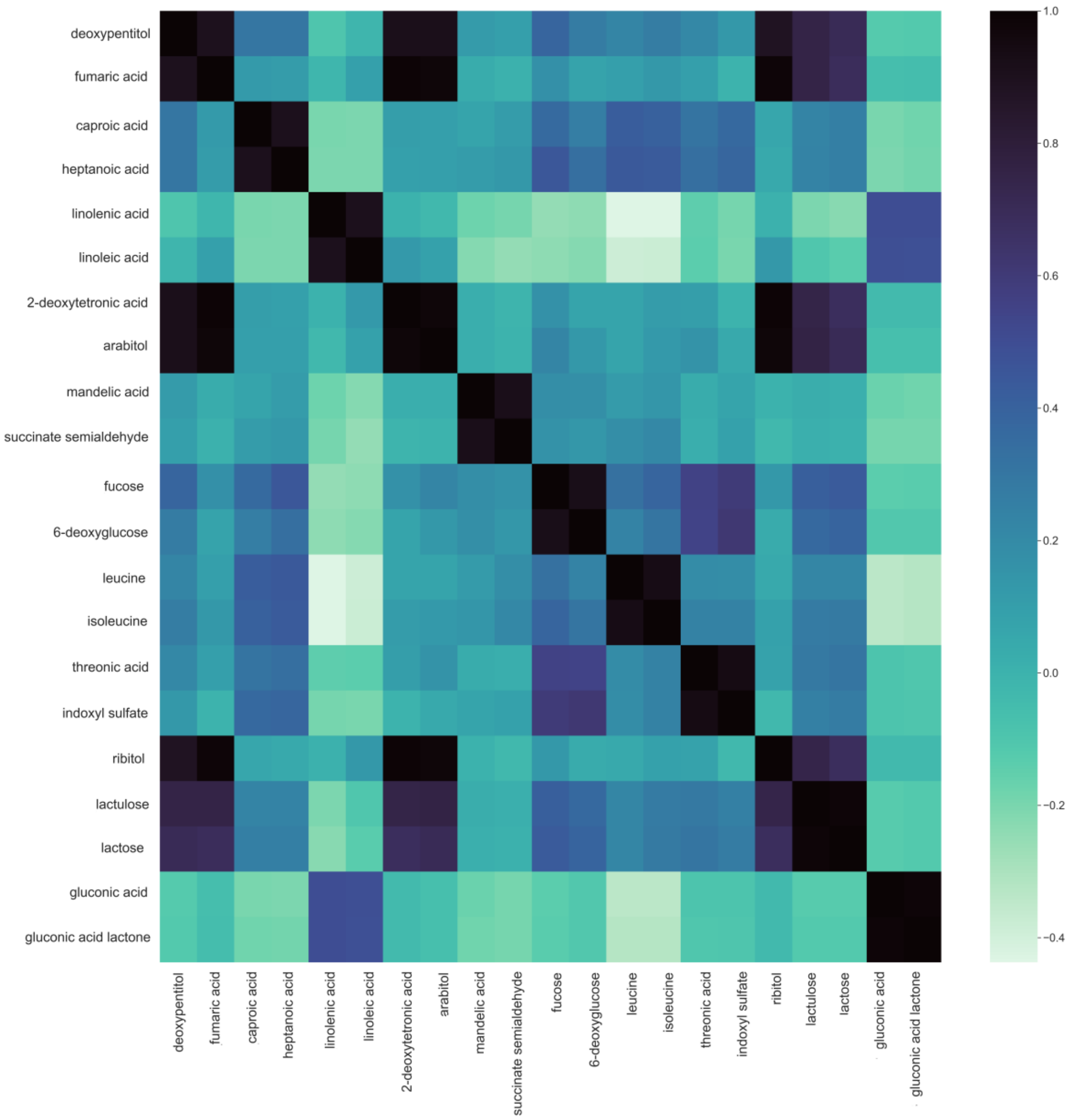

<u>Supplementary Figure S1</u>: Heatmap of Pearson's correlation coefficients a between plasma concentrations of metabolites with cut-off r>0.90. Significance for pairwise comparison detailed in the results section. The correlation score can be visualized through the scale bar on the right.

A

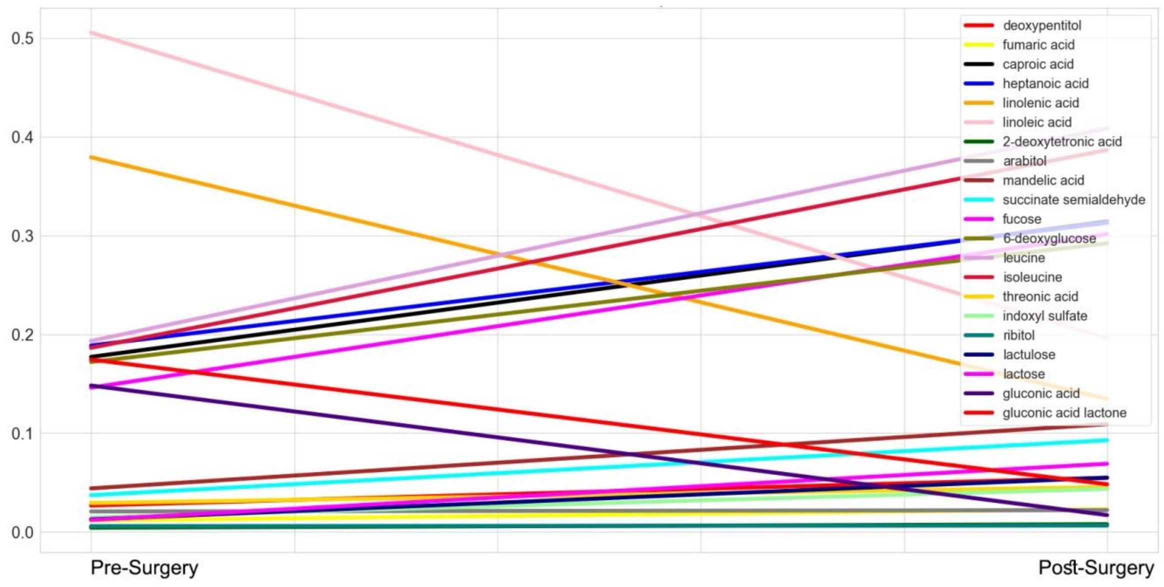
0.5
0.4
0.3
0.2
0.1
0.0
Pre-Surgery
Post-Surgery
deoxypentitol
fumaric acid
caproic acid
heptanoic acid
linolenic acid
linoleic acid
2-deoxytetronic acid
arabitol
mandelic acid
succinate semialdehyde
fucose
6-deoxyglucose
leucine
isoleucine
threonic acid
indoxyl sulfate
ribitol
lactulose
lactose
gluconic acid
gluconic acid lactone

B

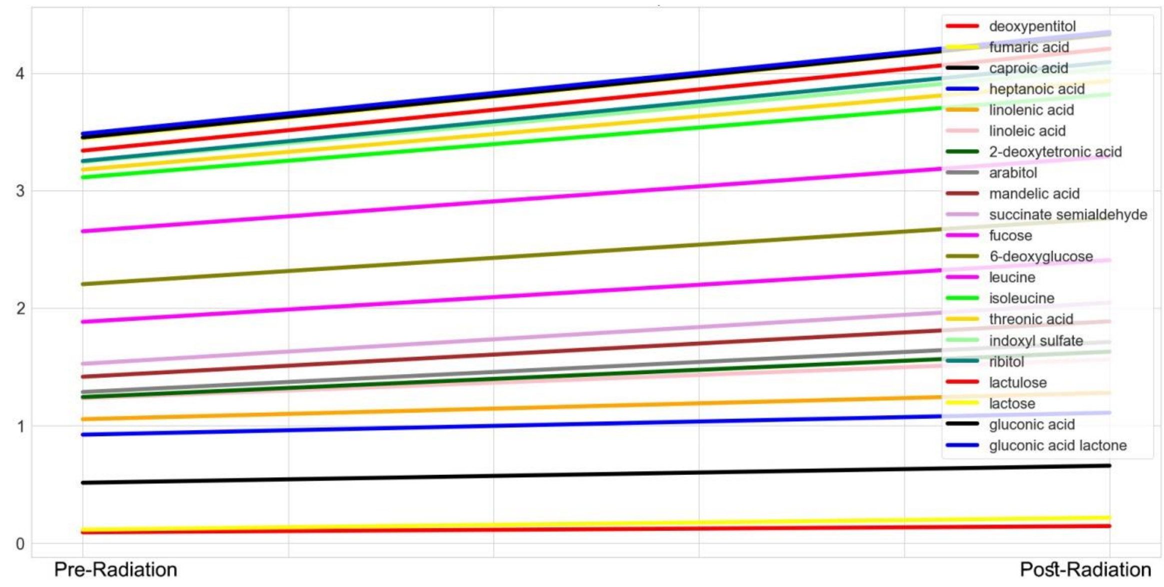
4
3
2
1
0
Pre-Radiation
Post-Radiation
deoxypentitol
fumaric acid
caproic acid
heptanoic acid
linolenic acid
linoleic acid
2-deoxytetronic acid
arabitol
mandelic acid
succinate semialdehyde
fucose
6-deoxyglucose
leucine
isoleucine
threonic acid
indoxyl sulfate
ribitol
lactulose
lactose
gluconic acid
gluconic acid lactone

C

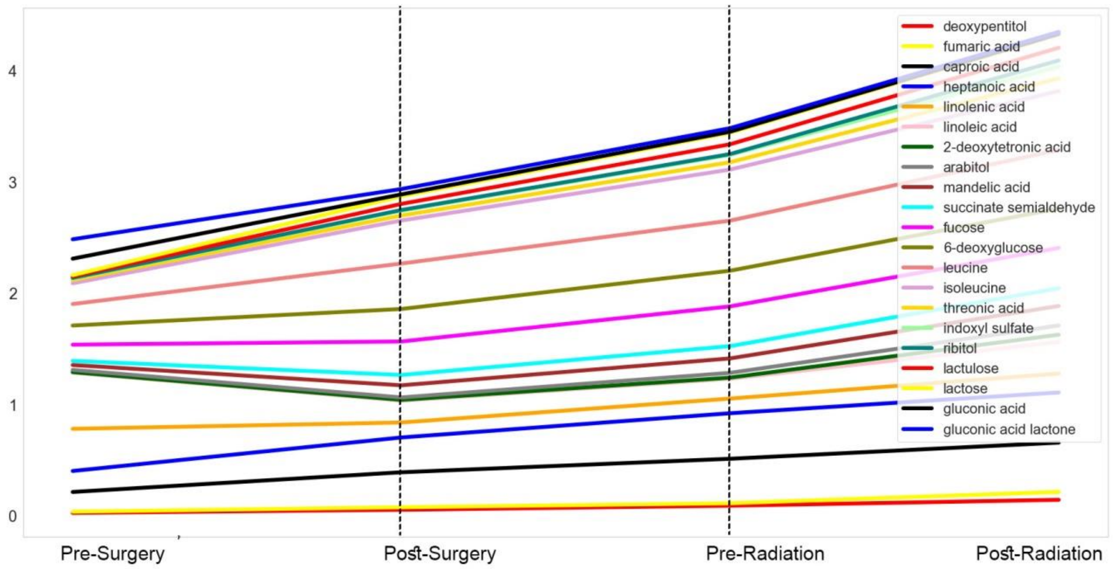

<u>Supplementary Figure S2:</u> Comparative profiling of highly correlated metabolites per treatment stage. The lines represent mean of the scaled data for each metabolite. **A**: Comparison of levels of highly correlated metabolites in pre-surgery vs post-surgery. **B**: Comparison of levels of highly correlated metabolites in pre-radiation vs post-radiation. **C**: Comparison of levels of highly correlated metabolites in the four stages of treatment.
